# Immunosuppression of the Nasal Cavity by a Novel Pathogenic Pseudorabies Virus Isolation from Cattle in China

**DOI:** 10.1155/2024/9652297

**Published:** 2024-06-21

**Authors:** Jian Zheng, Mei Fu, Zhiyi Yin, Zhi Dou, Jian Lin, Guangjun Chang, Qian Yang

**Affiliations:** ^1^ MOE Joint International Research Laboratory of Animal Health and Food Safety College of Veterinary Medicine Nanjing Agricultural University, Nanjing, Jiangsu 210095, China; ^2^ Ministry of Education Joint International Research Laboratory of Animal Health and Food Safety College of Veterinary Medicine Nanjing Agricultural University, Nanjing, Jiangsu 210095, China

## Abstract

The respiratory mucosa serves as a primary entry point for numerous pathogenic microbes, and the respiratory mucosa secretes type I and III interferons (IFNs), the first generation of antiviral cytokines, in response to viral infection. The pseudorabies virus (PRV) causes serious illnesses in many domestic and wild animal species, particularly in pigs and cattle. However, more information is needed about the immunosuppressive properties and evolutionary history of emerging PRV field strains in China's respiratory system. The PRV field strain JS2022, which was obtained from a cow farm for this investigation, is a spontaneous recombination of early PRV variant strains in the Jiangsu region and is similar to the PRV variations recovered in China in terms of its entire genome sequence. According to sequence analysis, JS2022 has a spontaneous deletion of 1,212 bp in the gE gene, 502 bp in the gI gene, and 192 bp in the glycoprotein (g) C gene. Pathogenicity analysis revealed that intranasal JS2022 causes severe neurological symptoms in calves, but this effect is different from that of ZJ01. In addition, a considerable number of viral antigens in the nasal mucosa were detected by immunohistochemical staining. Therefore, we constructed a bovine nasal mucosal explant model that maintained good cell morphology and activity even after 5 days. In bovine nasal mucosal explants, JS2022 and ZJ01 can cause infection, and the viral load increases dramatically over time. Quantitative research revealed that 24 hr after infection, JS2022 dramatically reduced the expression of downstream interferon-stimulated genes and the innate immune factors IFN-*β* and IFN-*λ*3 and bovine nasal mucosal explants. Overall, our results highlight the significance of PRV surveillance in cattle and offer a resource for learning more about the clinical traits and development of PRV.

## 1. Introduction

The virus that causes pseudorabies, sometimes referred to as Aujeszky's sickness, is the source of pseudorabies and has caused significant financial harm to the world's breeding business [1, 2]. Primarily affecting members of the Suidae family, PRV can also infect horses, dogs, cats, sheep, and goats, among other domestic and wild mammals. It typically results in the death of these hosts [3, 4]. Acute neurological symptoms, such as circular movement, pruritus, high temperature, dyspnea, and coma, are indicative of PRV infection in animals and are associated with increased mortality rates [5]. Since the 1970s, when China imported the Bartha-K61 vaccine from Hungary, the incidence rate and newborn piglet mortality in infected pig herds have dramatically decreased to less than 10% [4, 6]. However, since 2011, outbreaks of variant PRV have been reported at many large-scale aquaculture farms in China [7, 8, 9, 10]. The fact that more cases of PRV infection in humans have been documented in China since 2018 is especially notable since it suggests that there is a new animal-derived viral hazard to human health, especially in light of the appearance of new mutant strains.

The double-stranded DNA virus known as PRV is about 70 kb long and a member of the Herpesviridae family's Alpha-Herpesviridae subfamily. The interaction between heparan sulfate proteoglycans and glycoprotein (g) C in the extracellular matrix of permissive cells mediates the attachment process of PRV virions. PRV gD stabilizes virus–cell contact, which is another way it contributes to viral entry [11]. The primary PRV genes that determine the pathogenicity of infected neurons are thymidine kinase (TK), gI, and gE [12, 13, 14]. The sequencing of PRV is difficult because of the high GC content of the genome and the presence of repetitive repeat sequences [11]. Advances in sequencing technology, particularly the use of high-throughput sequencing, have led to the publication of numerous full PRV sequences in recent years [1, 15, 16]. At the moment, more thorough sequence data for the PRV strains are required. These data are required in order to offer a helpful reference for the analysis of the occurrence and development of PRV.

The respiratory mucosa is where PRV first replicates. From there, it travels via neurons, lymphatic vessels, and blood vessels to the central nervous system and internal organs, where it eventually results in disease [17]. In addition, special defensive mechanisms have evolved in the respiratory mucosa to prevent viral infection. Type I and type II interferons (IFNs), the initial antiviral cytokines produced by the respiratory system in response to viral infection, are produced prior to the activation of the adaptive immune system [18, 19]. Type III IFNs were once thought to be unnecessary, but research has since demonstrated that they are essential for guarding respiratory mucosal surfaces against pathogen assaults [20]. The primary function of type I and III IFNs is to increase the production of hundreds of interferon-stimulated genes (ISGs), including MX1, MX2, and IFIT3, which provide cells with antiviral defense. In the process of coevolution, PRV has created a number of ways to counteract the antiviral effects of IFNs [21, 22]. Several proteins have been shown to be involved in inhibiting IFN signaling, including UL50, which induces IFNAR1 degradation, and US3 and UL42, which interfere with the transcription of ISGs [23, 24, 25]. However, information regarding whether PRV inhibits type III and type Ⅰ IFN in the nasal mucosa is currently unavailable.

In this work, we have successfully isolated a novel PRV field strain from Jiangsu, China cattle ranches, and we have obtained the complete PRV genome sequence by high-throughput sequencing. Additionally, their genetic evolution was examined. The genetic evolution, in vivo pathogenicity, and innate immunity-inhibiting qualities of these strains were also examined in the nasal mucosa. Our research will help clarify the evolution of PRV in China and provide guidance for the clinical management of PRV infection.

## 2. Materials and Methods

### 2.1. Cells, Viruses, and Ethics Statement

Cells of Vero, PK15, and MDBK were grown at 37°C in an incubator with 5% CO_2_ using minimal necessary medium (MEM) supplemented with 10% fetal bovine serum (FBS) and penicillin–streptomycin (HyClone). All animal experiments were approved by the Institutional Animal Care and Use Committee of Nanjing Agriculture University and followed the National Institutes of Health guidelines for the performance of animal experiments.

### 2.2. Isolation and Identification of PRV Field Strains

A cow that was believed to be dying from a pseudorabies virus infection had its brain tissue removed. The samples (weight/volume = 1 : 2) were broken up into tiny pieces and aseptically ground in sterile PBS. After three cycles of freezing and thawing, the supernatants were collected by centrifugation at 5,000 rpm and 4°C for 10 min. The DNA was then extracted using a Virus Genomics DNA Isolation Kit (Tiangen Biotech Co., Ltd.). The manufacturer's instructions were followed. Using primer pairs (Table 1) that were directed toward the gB gene, the PRV-positive samples were detected by PCR. MDBK cells cultured in 6-well cell culture plates were inoculated with a confluent monolayer of PRV-positive supernatants. The supernatants were collected and replaced with DMEM containing 1% FBS after a 2-hr incubation period at 37°C. The infected MDBK cells' cytotoxic effects (CPEs) were seen twice a day. After being incubated for 48 hr at 37°C, the cell supernatants were collected and used to extract DNA. The PRV field strains were successfully isolated and, as was previously indicated, identified by PCR using this method.

### 2.3. Plaque Purification and Size Determination

The obtained viruses were serially diluted 10 times, and MDBK cells grown in 6-well plates were treated with the 10^4^–10^6^-fold dilutions that were produced. The cells were incubated in DMEM supplemented with 2% FBS and 1% (w/v) low melting point agarose (Cambrex, Inc., USA) after being incubated for 1 hr at 37°C and three PBS washes. After 2 to 3 days of incubation at 37°C, plaques were visible. To create a single viral clone, three of the viral plaques in the agarose were selected and resuspended in DMEM. After centrifugation, the supernatants were used to infect Vero cells. The viruses were then isolated and utilized for the plaque purification procedures previously described. Three rounds of plaque purification were performed in order to produce the purified virus. To evaluate the plaque sizes produced for each purified virus, Vero cells were infected with the same amount of either the isolated virus or ZJ01 strains [26], and the above-described procedure for the plaque assay was followed. The plaques were stained with 5% (w/v) crystal violet at 72 hr postinfection (hpi), and ImageJ software was used to estimate the plaques' diameters. The standard deviation and mean diameter were obtained from three different experiments.

### 2.4. Growth Curves of the Isolated PRV Strains

The PRV strain was injected at a multiplicity of infection (MOI) of 0.1 into Vero, PK15, and MDBK cells. Different times were used to collect the cell supernatants. Quantitative RT-PCR was used to determine the viral load at different time points. For every PRV strain, a multistep growth curve was created using the virus titer as the ordinate and the infection time as the abscissa.

### 2.5. High-Throughput Sequencing of the Complete PRV Genome

The full genomic DNA of the purified PRV strains was extracted using a Virus Genomics DNA Isolation Kit (DP315, Tiangen Biotech Co., Ltd.) in compliance with the manufacturer's instructions. By using high-throughput sequencing at Shanghai Personal Biotechnology Co., Ltd., the entire genome sequence of the isolated strains was identified.

### 2.6. Sequence Alignment and Phylogenetic Analysis

The complete sequences of the isolated strain obtained through high-throughput sequencing were opened in DNASTAR using EditSeq. The isolated bacteria's complete genomes and important virulence genes were compared to the PRV sequences from the NCBI, and the inferred amino acid sequences were aligned using the ClustalW program. The PRV whole-genome sequence (reference PRV whole-genome sequence, https://www.ncbi.nlm.nih.gov/genome/browse/#!/viruses/4631/) or the major immunogenic and virulence genes (gC, gD, gI, gG, and TK) were used to construct phylogenetic trees using the maximum likelihood (ML) method in Molecular Evolutionary Genetics (MEGA 7) software.

### 2.7. Recombination Analysis

Using Simplot software (version 3.5.1), recombination analysis of the isolated strains' full PRV genome was performed. Potential recombinant events were identified by moving in 200 nucleotide steps within a sliding window of 2,000 nucleotides.

### 2.8. Experimental Infection of Calves

Six calves (1-week-old) were randomly divided into two groups (*n* = 3 in each group). Ten milliliter of the JS2022 strain (10^7^ PFU/mL) was administered intraperitoneally to the JS2022 group, whereas 10 mL of DMEM was supplied to the negative control (NC) group as an uninfected control. Each group's clinical symptoms were observed every day for 6 days after vaccination. Following earlier reports, the brain tissues of calves that passed away during the observation period were processed for histological analyses after they were dissected [27]. After 6 days of vaccination, all calves in the NC group were allowed to sleep, and the brain tissues were removed and removed using sterile PBS. The tissues from different parts of the calves were collected for subsequent qPCR and histological analysis.

### 2.9. Isolation and Culture of Bovine Nasal Mucosal Explants

**Figure 1:**
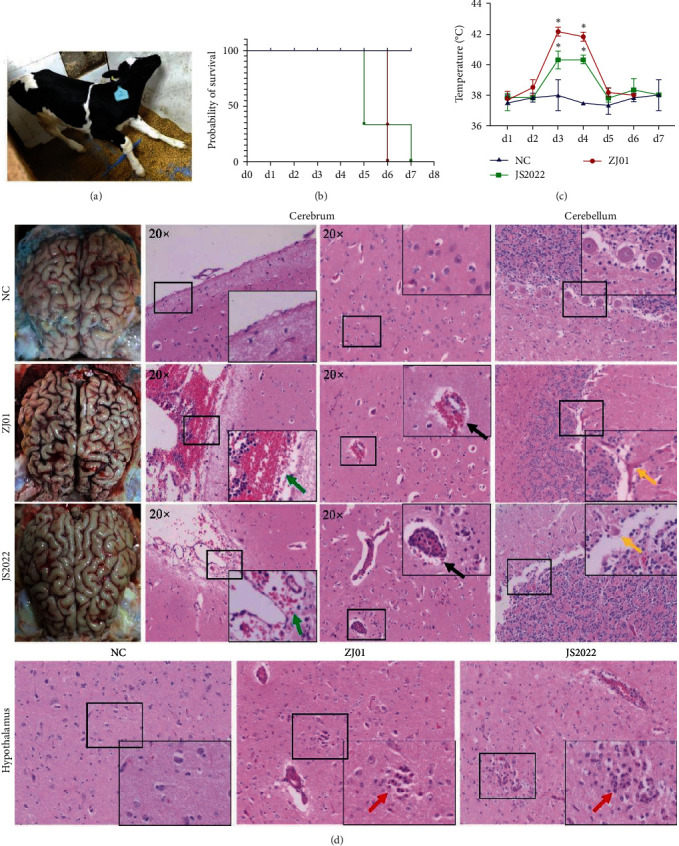
Clinical characteristics and pathological changes in mice challenged with the JS2022 and ZJ01 strains. (a) Calves infected with the JS2022 strain showed convulsions and ataxia before death. (b) Survival curve of calves after challenge. (c) The temperature of calves after challenge. (d) Gross and histopathological images of lesions in the cerebrum, cerebellum, and hypothalamus of infected cattle. The arrow points to the lesion site, indicating meningeal congestion (green arrows), mononuclear perivascular cuffing (black arrows), neuronal necrosis (yellow arrows), and inflammatory cell infiltration (red arrows).  ^*∗*^, *P* < 0.05.

The nasal conchae of three healthy bovine heads removed from slaughterhouses were submerged in PBS solution supplemented with 1% amphotericin, 5% streptomycin, and gentamicin. The tissue used for the bovine nasal mucosal explants was acquired using our earlier operating technique [28]. After being prepared, the nasal mucosal tissue was positioned with its mucosal surface facing upward on a 12-well transwell device. The pore diameter of the upper chamber filter membrane was 0.4 *μ*M. The entire transwell apparatus received about 600 *μ*L of culture medium [29], which is only 50% of the thickness of the mucosal tissue (Figure 1(a)). After that, the tissue was cultivated in 5% CO_2_ at 37°C, and fresh medium was added every day.

The cilia in the nasal mucosal explants were oriented outward due to folding at various periods during culture. After that, a video recorder was used to capture the folded position under an optical microscope. Samples of the bovine nasal mucosal explants were taken at various intervals for subsequent histological examination.

### 2.10. H&E and Immunohistochemical Staining

The samples were embedded in paraffin after being fixed for 24 hr in a 4% formaldehyde solution buffered with phosphate. The cut sections (5 mm thick) were rehydrated in descending alcohol grades, deparaffinized in xylene, and routinely stained with hematoxylin and eosin. The sections of each tissue were examined under a microscope.

Antigen retrieval using citrate buffer at pH = 6.0 was done in a Decloaking Chamber for 30 min at 95°C in order to undertake immunohistochemical analysis. Slides were blocked for 1 hr with 5% bovine serum albumin (BSA) and then incubated with anti-Claudin1 (1 : 200) or anti-gB (1 : 1,000) throughout the entire night at 4°C in a humidified atmosphere. The SABC-POD Kit was utilized for signal amplification and visualization.

### 2.11. Immunofluorescence

The sections were washed, as was previously indicated, and then exposed to antigen demasking and a 1-hr 5% BSA treatment. Next, mouse anti-PCNA (Abcam) was incubated overnight at 4°C, and fluorescent secondary antibody was incubated for 1 hr at 25°C. Using a confocal laser microscope (LSM-710; Zeiss), the cell nuclei were stained and studied following a 5-min incubation period with diamidino-2-phenylindole (DAPI).

### 2.12. Experimental Infection of Nasal Mucosal Explants

Following the placement of the nasal mucosal explants in a 24-well plate, the mucosal tissue was inoculated with PRV (5 × 10^5^, 400 *μ*L). After being cleaned three times in a single hour, the nasal mucosal tissue was transferred to a 12-well transwell device. The nasal mucosa was cultivated in 5% CO_2_ at 37°C. For further analysis, the infected nasal mucosa was removed at various intervals (0, 4, 12, 24, and 48 hr).

### 2.13. RNA Extraction, Quantitative Real-Time PCR, DNA Extraction, and qPCR

TRIzol reagent (Invitrogen) was used to extract total RNA from mucosal explants from the nasal cavity of the cows. Reverse transcription was used to generate cDNA using HiScript™ Q RT SuperMix for qPCR (R223-01, Vazyme, China). Target gene transcription was determined by quantitative RT-PCR (RT-qPCR) using the SYBR Green qPCR Kit (Takara, Beijing, China), and analysis was performed using the double standard curve method. All of the primers used for RT-qPCR are listed in Table 1. Glyceraldehyde 3-phosphate dehydrogenase (GAPDH), the internal control, was quantified. The 2^−*ΔΔ*CT^ method was utilized to ascertain the relative quantity of cytokine RNA.

Three randomly selected samples of organ tissue were obtained from various sections, each weighing 0.1 g, for quantitative analysis. The organ's viral load was then calculated as the mean of the test results. The viral DNA of the infected tissues was extracted using technique 2.5. For every experiment, a standard curve was produced via coamplification with known quantities of the PRV gB plasmid. Using a tenfold dilution, the gB standard plasmid was employed as a qPCR template. Next, using the Ct values of standard plasmids at various concentrations, a standard curve was created. In order to ascertain the corresponding copy number of the virus genomic DNA, the sample's Ct value is finally incorporated into the standard curve. The following sequence was used for the fluorescence probe: 5′-FAM-CCGCGTACGTGCTCGGACCA-BHQ1-3′.

### 2.14. Statistical Analysis

At least three trials were conducted for each experiment. The data are displayed as the mean ± standard deviation (SD). GraphPad Prism version 7.0 was used for the statistical analysis. To assess statistically significant differences between different groups, a one-way analysis of variance (ANOVA) or a *t*-test was used. The figures' statistical significance is shown as follows:  ^*∗*^, *P* < 0.05.

## 3. Results

### 3.1. Isolation and Biological Characteristics of the PRV Field Strains

**Figure 2:**
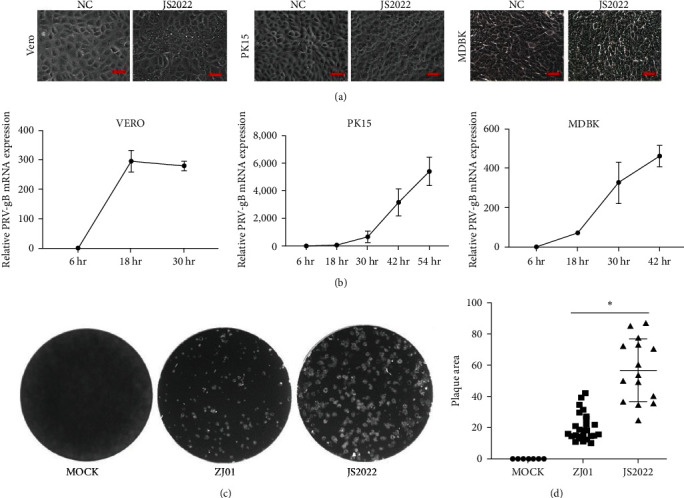
Biological characteristics of the isolated field JS2022 strains. (a) Vero, PK15, and MDBK cells were inoculated with the PRV JS2022 strain or supplemented with MEM as a negative control, and the cytopathic effects were observed via light microscopy. (b) Multistep growth curves of the JS2022 strain in Vero, PK15, and MDBK cells. (c) Vero cells were infected with the JS2022 and ZJ01 strains. The plaque size of each virus was assessed by performing a plaque assay as described in the section Materials and Methods. (d) Statistical chart of plaque size. Bar graphs displaying the means ± SDs. Statistically significant differences are indicated by  ^*∗*^, *P* < 0.05.

Vero, PK15, and MDBK cells were inoculated using the supernatants of the PRV-positive tissues found by PCR. Within 48 hr following inoculation, two cells infected with tissue supernatant exhibited characteristic CPEs, such as tissue aggregation, rounding, and removal, along with the development of vacuole-like lesions. Conversely, negative cells that were given MEM containing 2% FBS during the observation period seemed normal (Figure 2(a)). Additionally, PCR was employed to validate the effective isolation of PRV field strains from tissue samples; these strains were designated as JS2022. The multistep growth curve (Figure 2(b)) showed that the two PRV strains isolated from the JS2022 strain exhibited similar growth properties in Vero, PK15, and MDBK cells.

The measurement of growth curves and plaque sizes investigated the biological characteristics of the isolated strains. The ZJ01 strain had a much larger mean plaque diameter than the JS2022 strain, as indicated by Figure 2(c) (*P* < 0.05).

### 3.2. Genetic Characteristics and Phylogenetic Analysis of the JS2022 Strain

High-throughput sequencing techniques were used to obtain the whole-genome sequence of the JS2022 strain to determine its genetic properties. The whole genome of the JS2022 strain measured 1,140,547 bp. The genomic architecture of these strains is identical to that of other known PRV strains, and the JS2022 strain contains 75 open reading frames (ORFs). JS2022 had a GC content of 73.63%.

**Figure 3:**
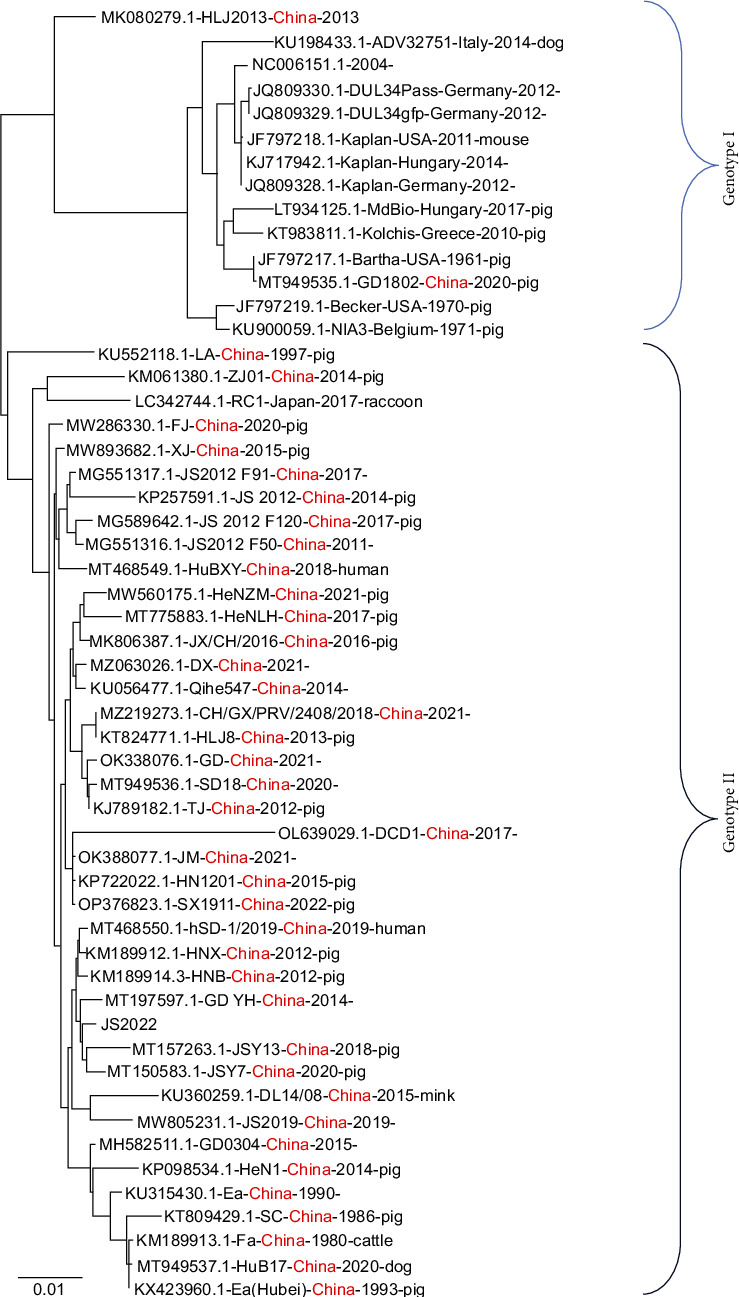
Phylogenetic analysis of the complete PRV genome sequence. A phylogenetic tree based on the complete genome sequence of PRV was constructed using the maximum likelihood (ML) method.

To examine the phylogenetic relationships between PRV strains, we created a maximum likelihood (ML) phylogenetic tree using 39 strains whose full-length sequences were obtained from the NCBI. These findings demonstrated that two groups can be formed phylogenetically from all the PRV strains. Nearly all strains isolated from China were grouped with foreign strains in a separate group (Genotype Ⅱ), while all strains exported from China were grouped in the same group (Genotype Ⅰ). Interestingly, the PRV GD1802 and HLJ2013 strains were grouped with strains from other nations. The PRV variants GD-YH and JSY13 were more closely related to JS2022, which was grouped in Genotype II (Figure 3).

### 3.3. Phylogenetic Analysis Based on the Major Immunogenic and Virulence Genes of JS2022

**Figure 4:**
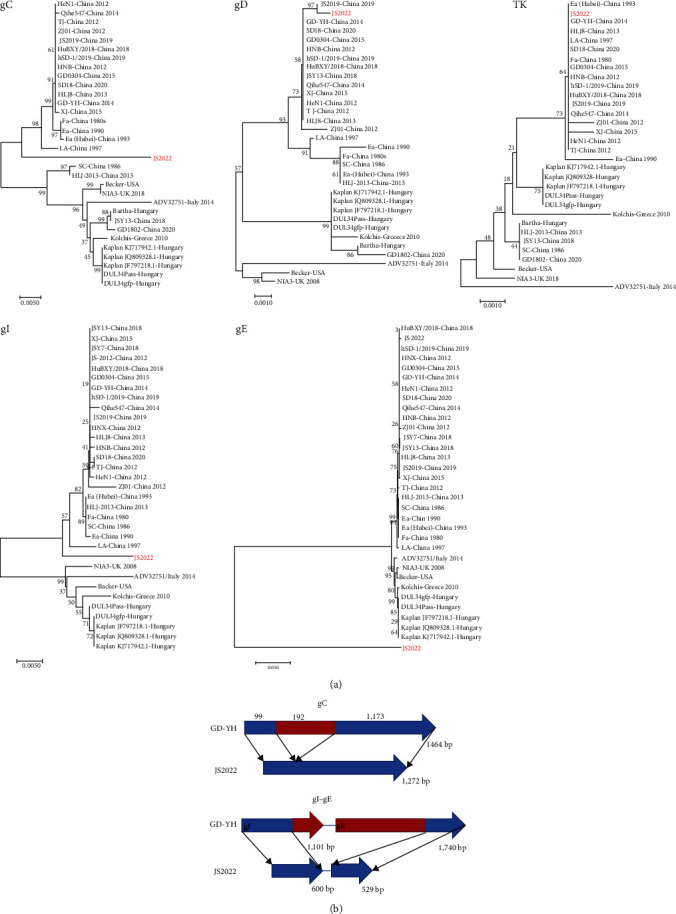
Phylogenetic analysis based on the major PRV immunogenic and virulence genes. (a) Phylogenetic trees based on the gC, gD, TK, gI, and gE genes were constructed by using MEGA7 software with the neighbor joining tree method. (b) We drew a pattern map of the gC, gI, and gE genes in JS222, with red representing missing fragments.

To further investigate the genetic development of the important immunogenic and virulent PRV genes, phylogenetic trees were built using the gC, gD, TK, gI, and gE genes as the bases. The findings demonstrated that the gD and TK genes for the JS2022 strain were grouped with the PRV variations that were identified in China, which were represented by the GD-YH strain. The GD-YH strain, which is representative of the PRV variations discovered in China, was shown to cluster with gC and gI, but it developed into a separate branch. The various classifications of the PRV strains do not depend on the gE gene. After further investigation, we discovered that the gC gene of JS2022 continuously lost 192 bp compared to that of the GD-YH strain. Furthermore, in contrast to the GD-YH strain, the gI gene exhibited a continuous deletion of 501 bp at the end, but the gE gene lacked a significant portion at the beginning of 1,211 bp (Figure 4).

### 3.4. Recombinant Analysis of Strain JS2022

**Figure 5:**
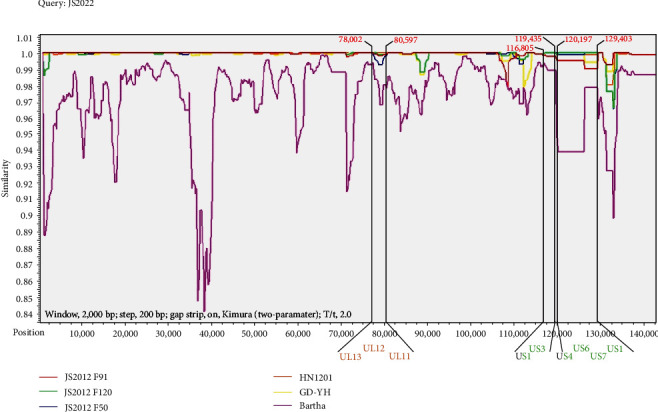
Recombination analysis of the JS2022 strain. Simplot software was used for the analysis of recombinant events in the complete genome of strain JS2022. Similarity analysis was performed with the query strain (JS2022) and the comparison strains (JS2012 F91, JS2012 F120, JS2012 F50, HN1201, GD-YH, and Bartha). The analysis was performed with the following parameters: window, 2,000 bp; step, 200 bp; gap strip, on, Kimura (two-parameter); and T/t, 2.0. Alignment of the ORFs in the recombination regions. The orange ORF indicates that the J2022 ORF was 100% similar to that of the HN1201 strain, the green ORF indicates 100% similarity to that of the JS2012 F120 strain, and the black ORF indicates overlap.

The JS2022 strain may be a recombinant strain, according to phylogenetic analysis based on the primary PRV genes for virulence and immunogenicity. Recombinant analysis was performed using the Simplot tool to find additional putative recombinant events that occurred over the whole genome of JS2022. The majority of the JS2022 sequences, as depicted in Figure 5, are consistent with those of JS2022 F91 and may include three putative recombination sites. The findings indicated that US3, US6, US7, and US1 were identical to the JS2012 F120 strain, and that 16 consensus ORFs of JS2022 from UL13 to UL11 were the same as those of the HN1201 strain. Partially consistent with the JS2022 sequence are US1 and US4. The conclusion that JS2022 may be a naturally occurring recombinant strain between the minor parental JS2012 F120 strain and the major parental JS2012 F91 strain is supported by these data. Furthermore, we found no indication of a possible recombination zone in the Bartha strain.

### 3.5. Clinical Characteristics and Pathological Changes in Calves Infected with the JS2022 and ZJ01 Strains

To evaluate the virulence of the JS2022 strain, 10^8^ doses of either the PRV strain or DMEM were intranasally injected into 1-week-old calves, and each group's clinical signs were tracked every day for 7 days. After the challenge with JS2022, the calves presented various neurologic symptoms, including salivation, purulent nasal fluid around the nasolabial mirror, inability to stand up, convulsions, and ataxia. The ZJ01 group exhibited salivation, shortness of breath, ataxia, inability to stand up, mental fatigue, and loss of appetite (Figure 1(a)). The JS2022 group started to die on the sixth days postinfection, while the ZJ01 group started to die on the fifth days following the emergence of the clear clinical symptoms previously indicated (Figure 1(b)). After being challenged with the JS2022 and ZJ01 strains, calves exhibited a marked increase in body temperature on the second and third day (Figure 1(c)). The histological findings revealed leptomeningeal hyperemia and nonsuppurative encephalitis in the brains of the deceased calves, along with neuronal necrosis, mononuclear perivascular cuffing, and inflammatory cell infiltration (Figure 1(d)).

### 3.6. Virus Localization after Challenge with the JS2022 and ZJ01 Strains

**Figure 6:**
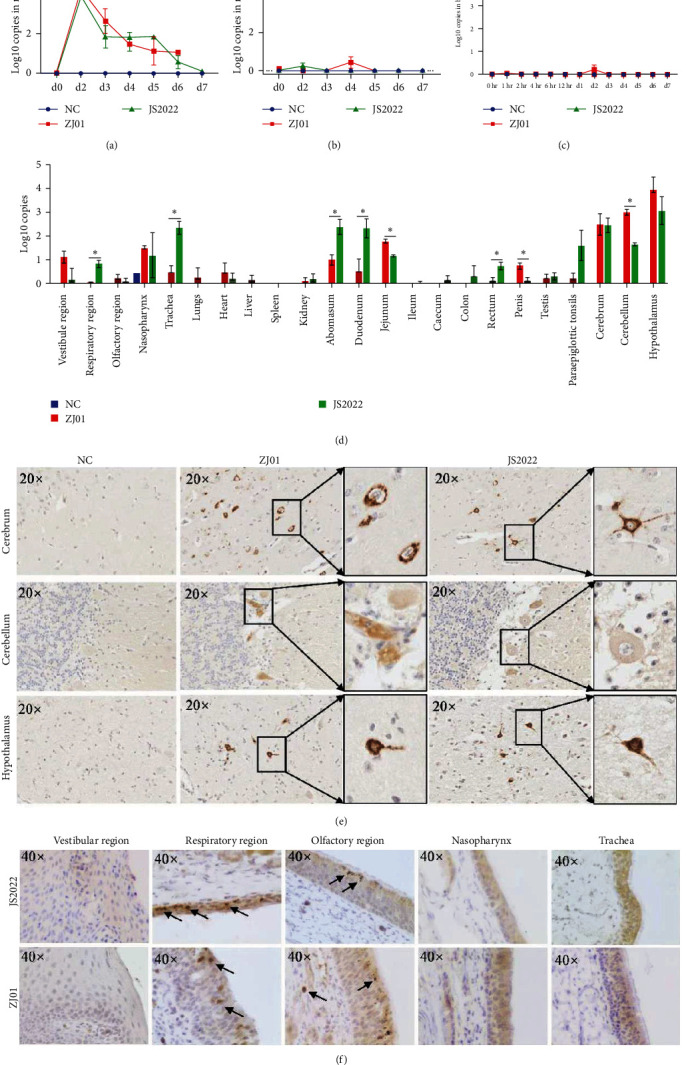
Virus localization after challenge with the JS2022 and ZJ01 strains. (a–c) PRV DNA from nasal swabs anal swabs and blood were detected by qPCR at different times after the challenge. (d) Detection of PRV DNA in different tissues of dead calves during the observation period via qPCR. (e and f) Immunohistochemistry (IHC) analysis of the cerebrum, cerebellum, hypothalamus, nasal cavity (vestibular, respiratory, and olfactory region), nasopharynx, and trachea with a mouse anti-PRV gB monoclonal antibody and brown positive particles (black arrows).  ^*∗*^*P* < 0.05.

We found that JS2022 and ZJ01 continued to exist in the nasal cavity after intranasal inoculation, but their loads significantly decreased over time (Figure 6(a)). However, no viruses were detected in the feces or blood throughout the entire infection cycle after intranasal inoculation (Figures 6(b) and 6(c)). In addition, the qPCR results revealed that a large number of viruses were present in the cerebrum, cerebellum, and hypothalamus of the calves (Figure 6(d)). The cerebrum, cerebellum, and hypothalamus were the primary locations of viral antigens, according to the results of the IHC analysis (Figure 6(e)). Additionally, viral antigens were found in the ZJ01 group's lungs as well as the abomasum and jejunum of the JS2022 strain (Figure S1(a)). In addition, we did not detect the antigen of PRV in the heart, liver, spleen, kidney, duodenum, ileum, cecum, colon, rectum, penis, testes, or para epiglottic tonsils in the JS2022 and ZJ01 groups (Figure S1(a)). Consistent with the qPCR results, the statistical results of the IHC images indicated that there was a greater content of viral antigens in the brain tissue and nasal mucosa (Figure S1(b)). The respiratory tract is a critical location for the invasion and infection of PRV. In this investigation, we discovered that the nasal cavity's olfactory and respiratory mucosa had a significant amount of viral antigens (Figure 6(f)).

### 3.7. Effect of the JS2022 Strain on Innate Immunity in Bovine Nasal Mucosal Explants

**Figure 7:**
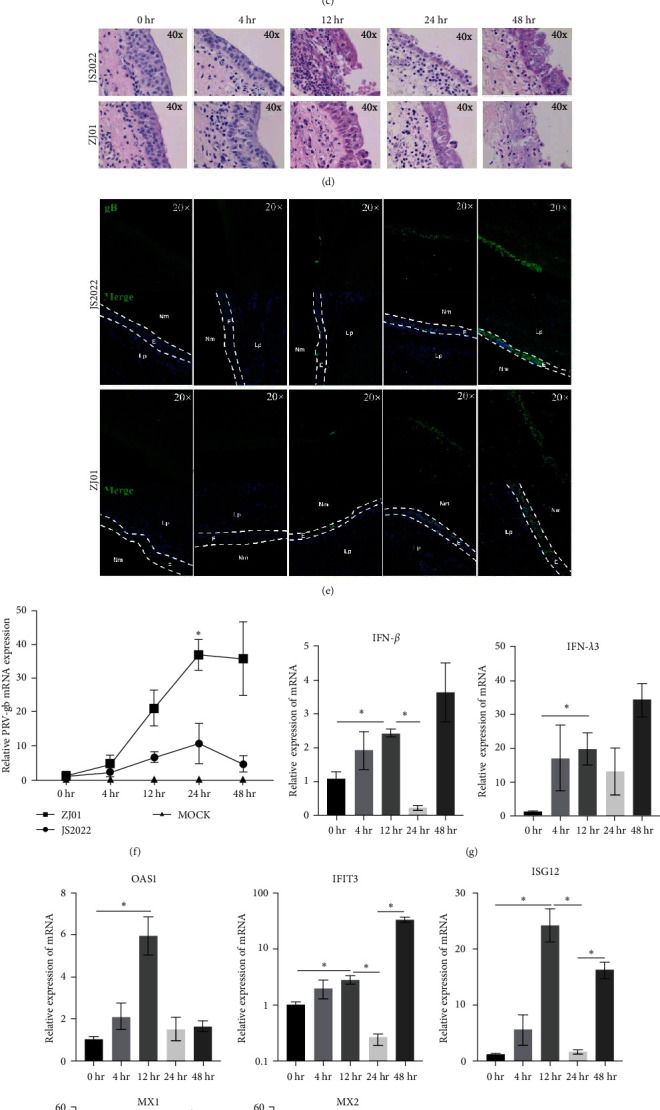
Characteristics of bovine nasal mucosal explants infected with JS2022. (a) Construction of the air–liquid culture model of bovine nasal mucosa explants. (b) H&E staining for the evaluation of structural characteristics. (c) Mucosal explants were inoculated with PRV JS2022 or ZJ01 for 1 hr, washed three times, and cultured for 0, 4, 12, 24, or 48 hr to collect samples. (d and e) H&E staining was used to evaluate the pathological characteristics of bovine nasal mucosal explants infected with JS2022 and ZJ01 at different times (0, 4, 12, 24, and 48 hr), and immunofluorescence was used to evaluate the distribution of JS2022 and ZJ01 in bovine nasal mucosal explants at different times (0, 4, 12, 24, and 48 hr). (f) Multistep growth curves of the JS2022 and ZJ01 strains in bovine nasal mucosal explants at different time points (0, 4, 12, 24, and 48 hr). (g and h) Quantification of the expression of interferon and interferon-stimulating genes in bovine nasal mucosal explants after infection with JS2022 at different times (0, 4, 12, 24, and 48 hr). E, epithelium; Lp, lamina propria; Nm, nasal meatus. Bar graphs displaying the means ± SDs.  ^*∗*^, *P* < 0.05.

To investigate PRV invasion and infection features in the nasal cavity, we established a culture model using explants of bovine nasal mucosa. For future experimental studies and real-world applications, the integrity and vitality of nasal mucosal explants are essential. The cilia of the explants of bovine nasal mucosa began to vibrate quickly after about 5 days. The bovine nasal mucosa exhibited a full histological structure and a well-preserved epithelial structure, as shown by the H&E staining data (Figure 7(b)). Then, we observed that the tight junction protein Claudin1 was mainly expressed in the epithelial layer of bovine nasal mucosal explants (Figure S2(a)). Finally, the results of the immunofluorescence staining demonstrated that the PCNA-positive cells were primarily found on the basal side of the epithelial layer of the bovine nasal mucosa and that the presence of these cells persisted until the fifth culture day (Figure S2(b)). These results indicate that the bovine nasal mucosal explants cultured in this study exhibited good integrity and vitality.

We inoculated bovine nasal mucosal explants with JS2022 and ZJ01 (Figure 7(c)). Following the inoculation of the JS2022 and ZJ01 strains into bovine mucosal explants, H&E staining revealed that the epithelial layer started to shed at 48 hr and the mucosal epithelium nuclei were vacuolated at 24 hr. The epithelial layer was damaged at 12 hr (Figure 7(d)). The immunofluorescence results revealed virus infection in the epithelial layer at 12 hr. The infected area continued to expand to the entire mucosal upper cortex over time (Figure 7(e)). Furthermore, using quantitative RT-PCR, we discovered that the viral load in the bovine nasal mucosa increased progressively throughout the culture period. At 24 hr, the viral load of JS2022 was noticeably greater than that of ZJ01 (Figure 7(f)).

We detected alterations in type I and III IFNs as well as the interferon boosting gene in bovine nasal mucosal explants, suggesting that the interferon system plays a significant role in preventing viral propagation. The results indicated that the expression levels of IFN-*β* and IFN-*λ*3 significantly increased at 12 hr compared to those at 0 hr but showed a downward trend at 24 hr. Surprisingly, the expression of IFN-*β* and IFN-*λ*3 showed an upward trend again after 48 hr. Moreover, the changes in downstream ISGs such as OAS1, IFIT3, ISG12, MX1, and MX2 were consistent with the changes in IFN-*β* and IFN-*λ*3 (Figures 7(g) and 7(h)).

## 4. Discussion

The respiratory tract is where PRV first replicates. From there, it travels via neurons, lymph, and blood vessels to the central nervous system and internal organs, including the kidney, lungs, and intestinal tract. Since 2011, a large number of PRV variations have been found in pig farms that received Bartha-K61 vaccination, significantly harming the bottom line of the Chinese pig industry [8, 9]. However, the introduction of numerous mutant strains has also resulted in deadly infections in other cattle due to the multihost infection features of PRV. In this instance, the JS2022 PRV field strain was isolated from cattle farms that may have been infected with PRV. Multistep growth curve data showed that in Vero, PK15, and MDBK cells, the JS2022 strain developed high titers. In particular, the JS2022 strain's mean plaque diameter was significantly higher than ZJ01′s. These results showed that the former was more susceptible to infection than the latter.

The genetic characteristics of the JS2022 strain were further examined by high-throughput sequencing. The findings demonstrated that the genome length, genome structure, and GC content of the JS2022 strain were comparable to those of previously obtained PRV strains [15, 30]. Based on the PRV full-length sequence, the Chinese new mutant strain and the JS2022 strain were clustered together, indicating that the PRV strain discovered in cattle is one of the many PRV mutant strains that have surfaced since 2011. Further investigation of the genetic evolution of the JS2022 strain involved the construction of phylogenetic trees based on the TK, gC, gD, gI, and gE genes. The findings demonstrated that the gE gene is independent of all other categories, whereas the gC and gI genes clustered with the previous Chinese classical strain, and the gD and TK genes clustered with the PRV variations identified in China. We hypothesized that the JS2022 strain might be a recombinant PRV strain as a result of this event. The evolution of the virus depends on natural recombinants between and within its genome, which can change the pathogenicity and ecology of the virus. Numerous PRV recombinant strains have been discovered in recent years. For example, spontaneous recombination between PRV genotype I of wild boars in China and genotype II of farmed pigs produced the PRV FJ62 variant that was discovered in Sichuan Province [31]. Two PRV variant strains, GXLB-2015 and GXGG-2016, were discovered in a swine farm that had received Bartha-K61 vaccination in 2023. A spontaneous hybrid between the Bartha strain and PRV variations produced the GXLB-2015 strain [32]. Three probable recombination sites in this investigation, which include the US1, US3, UL4, US6, and US7 genes, as well as UL13, UL12, UL11, and US1, all demonstrated strong similarity with the PRV variants recovered in China. According to our findings, JS2022 may be a naturally occurring recombinant strain among the PRV variants that were discovered in China. Since the majority of the recombination-involving strains are located in the Jiangsu region, multiple local strains of the pseudorabies virus likely recombined to create JS2022.

To test the virulence of JS2022, we intranasally administered JS2022 to calves. The results demonstrated that JS2022 exhibits strong virulence, with calf onset and death occurring 6 days after challenge, which is consistent with the death time of pigs after intranasal administration of PRV TJ [33]. Moreover, JS2022 results in significant neurological symptoms, which is in line with previous findings [34]. Severe respiratory disease is an important manifestation of PRV infection, and studies have shown significant pulmonary edema after intranasal administration of PRV [33]. However, in our study, the calves did not experience shortness of breath after the challenge with JS2022, and no obvious lung lesions were found during the autopsy.

In contrast, the calves inoculated intranasally with ZJ01 showed significant shortness of breath, and there were obvious lung lesions. IHC also revealed the presence of a large number of viral antigens in the lungs of the ZJ01 group, indicating the difference in virulence among different strains of PRV. The primary virulence proteins that allow PRV to infiltrate the human nervous system are the gI and gE glycoproteins. Trigeminal and olfactory nerve terminal invasion is prevented, and virulence is markedly reduced when the gI and gE genes are deleted [35, 36, 37]. In the present study, the JS2022 strain had a 502 bp deletion in the gI gene and a 1,212 bp deletion in the gE gene. Nonetheless, a large number of virions were still detected in the cerebrum, cerebellum, and hypothalamus after intranasal administration of the JS2022 strain. Therefore, the deletion sequences of the JS2022 gI and gE genes did not affect their propagation in the brain nerves, which also indicates that the first 500 bp of the gI gene and the last 529 bp of the gE gene are key sequences for PRV virulence.

Because the mucosal explant model preserves the connections between different cells and replicates the entire mucosal structure in vivo, it is suitable for in vitro etiological research [38, 39, 40, 41]. Previous studies have constructed a culture model of bovine nasal mucosal explants in which structural integrity and viability are maintained for at least 3 days [42]. Subsequently, Lennert Steukers et al. constructed a 4-day culture model for nasal mucosal explants [43]. In this work, we have effectively created a model of bovine nasal mucosal explant culture that, in air interface culture, retains substantial vitality and structural integrity for at least 5 days. As a result, our findings use the principles of reduction, refinement, and replacement (3R) and produce a homologous model that closely mimics the in vivo environment.

Bovine respiratory mucosal explants have been used to study bovine respiratory pathogen infections, including bovine herpesvirus-1, bovine pleuropneumonia, and influenza D viruses [38, 41, 43]. In this study, JS2022 successfully infected the nasal mucosa of bovines, indicating that PRV can infect cattle through the respiratory tract and revealing the multihost infection characteristics of PRV. Furthermore, the virus was initially detected in the respiratory mucosal explants' epithelium, which is in line with the outcomes of PRV inoculation in pig respiratory mucosal explants [39, 44, 45]. Furthermore, previous studies investigating the infection kinetics of many virus strains in pig respiratory mucosal explants shown that older strains, including NS374, Becker, and/or NIA1, were less able to form plaques and/or penetrate the basement membrane [39]. Due to the origin of JS2022 in bovines, we compared the infection characteristics of the highly virulent PRV strain ZJ01 from pigs [46] in the nasal mucosa of bovines. Compared with ZJ01, JS2022 exhibited a greater replication rate in bovine nasal mucosal explants. Therefore, JS2022 may be a PRV strain that is more adaptable to bovine hosts.

Important antiviral cytokines that guard against viral infections on local mucosal surfaces include type I and III IFNs [20]. PRV uses multiple strategies to inhibit type I and III IFN-induced signaling from evading host innate immunity [22, 47, 48, 49]. One of the research's limitations is that, instead of analyzing type I and III IFNs in vivo, the analysis focused solely on their expression levels in cells. PRV JS2022 was used in this investigation to inoculate bovine nasal mucosal explants, and the result was a considerable suppression of early type I and type III IFN production at 24 hr. These results, however, do not quite align with the results of the PRV strain Kaplan infection of intestinal pig epithelial cells, PK-15 cells, and porcine epithelial cells [21]. This could be caused by the various strains that were used, but it could also be attributed to the variations between the cell model and the explant model. It is interesting to note that at 48 hr, type I and III IFN expression levels dramatically rose once more. Due to the destruction of the epithelial layer by the virus at this stage, we speculate that other natural immune pathways or receptors in bovine nasal mucosal explants are activated, such as endogenous damage-related molecular patterns.

## 5. Conclusion

In this study, a natural recombinant strain between early PRV variant strains in the Jiangsu region, known as JS2022, was identified as a PRV field strain. JS2022 successfully established infection in bovine nasal mucosal explants and significantly inhibited the innate immune response. Our findings will serve as a guide for a deeper understanding of the immunosuppressive effects of PRV on the respiratory system and its evolution in China.

## Figures and Tables

**Table 1:** Primer list.

Gene	Forward	Reverse	Species
GAPDH	GGTCACCAGGGCTGCTTTTA	CCAGCATCACCCCACTTGAT	Bovine
IFN-*β*	AGAAGCAAAACACCACAGCG	CAGTCACGGACGTAACCTGT	
IFN-*λ*3	AGCCTTCAACGACTGATGCT	GAGGATATGGTGCAGGGTGT	
OAS1	GAAATGGGATGGGTCTCCCG	CCAAGCCCCCTATCCCTAGA	
IFIT3	AAGGCTGGGAAAGCAGATCC	CTGGAGAACGGAATGCCTCA	
ISG12	TGGTGCTGGTCAGTTTTGGT	AGAGTGAGCCCTCCTACTGG	
MX1	AGAGCAACCTGTACAGCCAA	GCCTCAGCACAAGAGGACAT	
MX2	CTACAAGTGCACAGGTGACAA	CGGGCACAGAACACAAAAGG	
GAPDH	TCATCATCTCTGCCCCTTCT	GTCATGAGTCCCTCCACGAT	Procine
GAPDH	ACATCATCCCTGCCTCTACTG	CCTGCTTCACCACCTTCTTG	*Chlorocebus aethiops*
gB	GTCCGTGAAGCGGTTCGTGAT	ACAAGTTCAAGGCCCACATCTAC	PRV

## Data Availability

The authors confirm that the data supporting the findings of this study are available within the article and its supplementary materials.

## Supplementary Materials

Supplementary Materials Figure S1: the localization of viral antigens in different organs of calves. Figure S2: the establishment of bovine nasal mucosal explant culture models.

## References

[1] Bo Z., Miao Y., Xi R. (2021). Emergence of a novel pathogenic recombinant virus from Bartha vaccine and variant pseudorabies virus in China. *Transboundary and Emerging Diseases*.

[2] Lee J. Y., Wilson M. R. (1979). A review of pseudorabies (Aujeszky’s disease) in pigs. *Canadian Veterinary Journal-Revue Veterinaire Canadienne*.

[3] Pomeranz L. E., Reynolds A. E., Hengartner C. J. (2005). Molecular biology of pseudorabies virus: impact on neurovirology and veterinary medicine. *Microbiology and Molecular Biology Reviews*.

[4] Tan L., Yao J., Yang Y. (2021). Current status and challenge of pseudorabies virus infection in China. *Virologica Sinica*.

[5] Sehl J., Teifke J. P. (2020). Comparative pathology of pseudorabies in different naturally and experimentally infected species—a review. *Pathogens*.

[6] Sun Y., Luo Y., Wang C.-H. (2016). Control of swine pseudorabies in China: opportunities and limitations. *Veterinary Microbiology*.

[7] An T.-Q., Peng J.-M., Tian Z.-J. (2013). Pseudorabies virus variant in Bartha-K61-vaccinated pigs,China 2012. *Emerging Infectious Diseases*.

[8] Gu J., Hu D., Peng T. (2018). Epidemiological investigation of pseudorabies in Shandong Province from 2013 to 2016. *Transboundary and Emerging Diseases*.

[9] Ma Z., Han Z., Liu Z. (2020). Epidemiological investigation of porcine pseudorabies virus and its coinfection rate in Shandong Province in China from 2015 to 2018. *Journal of Veterinary Science*.

[10] Tong W., Liu F., Zheng H. (2015). Emergence of a Pseudorabies virus variant with increased virulence to piglets. *Veterinary Microbiology*.

[11] Klupp B. G., Hengartner C. J., Mettenleiter T. C., Enquist L. W. (2004). Complete, annotated sequence of the pseudorabies virus genome. *Journal of Virology*.

[12] Ferrari M., Mettenleiter T. C., Romanelli M. G. (2000). A comparative study of pseudorabies virus (PRV) strains with defects in thymidine kinase and glycoprotein genes. *Journal of Comparative Pathology*.

[13] Husak P. J., Kuo T., Enquist L. W. (2000). Pseudorabies virus membrane proteins gI and gE facilitate anterograde spread of infection in projection-specific neurons in the rat. *Journal of Virology*.

[14] Xu L., Wei J.-F., Zhao J. (2022). The immunity protection of central nervous system induced by pseudorabies virus DelgI/gE/TK in mice. *Frontiers in Microbiology*.

[15] Bo Z., Li X. (2022). A review of pseudorabies virus variants: genomics, vaccination, transmission, and zoonotic potential. *Viruses*.

[16] Luo Y., Li N., Cong X. (2014). Pathogenicity and genomic characterization of a pseudorabies virus variant isolated from Bartha-K61-vaccinated swine population in China. *Veterinary Microbiology*.

[17] Wozniakowski G., Samorek-Salamonowicz E. (2015). Animal herpesviruses and their zoonotic potential for cross-species infection. *Annals of Agricultural and Environmental Medicine*.

[18] Lan F., Zhong H., Zhang N. (2019). IFN-lambda 1 enhances *Staphylococcus aureus* clearance in healthy nasal mucosa but not in nasal polyps. *The Journal of Allergy and Clinical Immunology*.

[19] Lopez J., Mommert M., Mouton W. (2021). Early nasal type I IFN immunity against SARS-CoV-2 is compromised in patients with autoantibodies against type I IFNs (vol 218, e20211211, 2021). *The Journal of Experimental Medicine*.

[20] Stanifer M. L., Guo C., Doldan P., Boulant S. (2020). Importance of type I and III interferons at respiratory and intestinal barrier surfaces. *Frontiers in Immunology*.

[21] Yin Y., Ma J. L., Van Waesberghe C., Devriendt B., Favoreel H. W. (2022). Pseudorabies virus-induced expression and antiviral activity of type I or type III interferon depend on the type of infected epithelial cell. *Frontiers in Immunology*.

[22] Yin Y., Romero Nás, Favoreel H. W., Longnecker R. M. (2021). Pseudorabies virus inhibits type I and type III Interferon-induced signaling via proteasomal degradation of janus kinases. *Journal of Virology*.

[23] Qin C., Zhang R., Lang Y. (2019). Bclaf1 critically regulates the type I interferon response and is degraded by alphaherpesvirus US3. *PLOS Pathogens*.

[24] Zhang R., Chen S. F., Zhang Y. (2021). Pseudorabies virus DNA polymerase processivity factor UL42 inhibits type I IFN response by preventing ISGF3-ISRE interaction. *The Journal of Immunology*.

[25] Zhang R., Xu A., Qin C. (2017). Pseudorabies virus dUTPase UL50 induces lysosomal degradation of type I interferon receptor 1 and antagonizes the alpha interferon response. *Journal of Virology*.

[26] Gu Z., Dong J., Wang J. (2015). A novel inactivated gE/gI deleted pseudorabies virus (PRV) vaccine completely protects pigs from an emerged variant PRV challenge. *Virus Research*.

[27] Song C., Huang X., Gao Y. (2021). Histopathology of brain functional areas in pigs infected by porcine pseudorabies virus. *Research in Veterinary Science*.

[28] Zheng J., Lin J., Ma Y. (2023). Establishment of sheep nasal mucosa explant model and its application in antiviral research. *Frontiers in Microbiology*.

[29] Alfi O., From I., Yakirevitch A. (2020). Human nasal turbinate tissues in organ culture as a model for human cytomegalovirus infection at the mucosal entry site. *Journal of Virology*.

[30] Ye C., Guo J.-C., Gao J.-C. (2016). Genomic analyses reveal that partial sequence of an earlier pseudorabies virus in China is originated from a Bartha-vaccine-like strain. *Virology*.

[31] Huang J., Zhu L., Zhao J. (2020). Genetic evolution analysis of novel recombinant pseudorabies virus strain in Sichuan, China. *Transboundary and Emerging Diseases*.

[32] Qin Y., Qin S., Huang X. (2023). Isolation and identification of two novel pseudorabies viruses with natural recombination or TK gene deletion in China. *Veterinary Microbiology*.

[33] Wang Y., Xia S.-L., Lei J.-L. (2015). Dose-dependent pathogenicity of a pseudorabies virus variant in pigs inoculated via intranasal route. *Veterinary Immunology and Immunopathology*.

[34] Cheng Z., Kong Z., Liu P. (2020). Natural infection of a variant pseudorabies virus leads to bovine death in China. *Transboundary and Emerging Diseases*.

[35] Johnson D. C., Webb M., Wisner T. W., Brunetti C. (2001). Herpes simplex virus gE/gI sorts nascent virions to epithelial cell junctions, promoting virus spread. *Journal of Virology*.

[36] Kratchmarov R., Kramer T., Greco T. M. (2013). Glycoproteins gE and gI are required for efficient KIF1A-dependent anterograde axonal transport of alphaherpesvirus particles in neurons. *Journal of Virology*.

[37] Polcicova K., Goldsmith K., Rainish B. L., Wisner T. W., Johnson D. C. (2005). The extracellular domain of herpes simplex virus gE is indispensable for efficient cell-to-cell spread: evidence for gE/gI receptors. *Journal of Virology*.

[38] Di Teodoro G., Marruchella G., Di Provvido A. (2018). Respiratory explants as a model to investigate early events of contagious bovine pleuropneumonia infection. *Veterinary Research*.

[39] Glorieux S., Favoreel H. W., Meesen G., de vos W., Van den Broeck W., Nauwynck H. J. (2009). Different replication characteristics of historical pseudorabies virus strains in porcine respiratory nasal mucosa explants. *Veterinary Microbiology*.

[40] Grivel J.-C., Margolis L. (2009). Use of human tissue explants to study human infectious agents. *Nature Protocols*.

[41] Mazzetto E., Bortolami A., Fusaro A. (2020). Replication of influenza D viruses of bovine and swine origin in ovine respiratory explants and their attachment to the respiratory tract of bovine, sheep, goat, horse, and swine. *Frontiers in Microbiology*.

[42] Niesalla H. S., Dale A., Slater J. D. (2009). Critical assessment of an in vitro bovine respiratory organ culture system: a model of bovine herpesvirus-1 infection. *Journal of Virological Methods*.

[43] Steukers L., Vandekerckhove A. P., Van den Broeck W., Glorieux S., Nauwynck H. J. (2012). Kinetics of BoHV-1 dissemination in an in vitro culture of bovine upper respiratory tract mucosa explants. *ILAR Journal*.

[44] Lamote J. A. S., Glorieux S., Nauwynck H. J., Favoreel H. W. (2016). The US3 protein of pseudorabies virus drives viral passage across the basement membrane in porcine respiratory mucosa explants. *Journal of Virology*.

[45] Pol J. M. A., Broekhuysendavies J. M., Wagenaar F., Labonnardiere C. (1991). The influence of porcine recombinant interferon-alpha-1 on pseudorabies virus-infection of porcine nasal-mucosa invitro. *Journal of General Virology*.

[46] Gu Z., Hou C., Sun H. (2015). Emergence of highly virulent pseudorabies virus in southern China. *Canadian Journal of Veterinary Research = Revue Canadienne De Recherche Veterinaire*.

[47] Bo Z., Miao Y., Xi R. (2020). PRV UL13 inhibits cGAS-STING-mediated IFN-*β* production by phosphorylating IRF3. *Veterinary Research*.

[48] Wang M., Liu Y., Qin C. (2022). Pseudorabies virus EP0 antagonizes the type I interferon response via inhibiting IRF9 transcription. *Journal of Virology*.

[49] Xie J., Zhang X., Chen L. (2021). Pseudorabies virus US3 protein inhibits IFN-beta production by interacting with IRF3 to block its activation. *Frontiers in Microbiology*.

