# Supplementary material for: Immunosuppression of the Nasal Cavity by a Novel Pathogenic Pseudorabies Virus Isolation from Cattle in China
Source: Transbound Emerg Dis. 2024 Jun 21;2024:9652297. doi: 10.1155/2024/9652297 (PMC12017080; doi:10.1155/2024/9652297)
Supplement: Supplementary Materials — Figure S1: the localization of viral antigens in different organs of calves. Figure S2: the establishment of bovine nasal mucosal explant culture models. [file 9652297.f1.docx]

**Supplementary Figures**


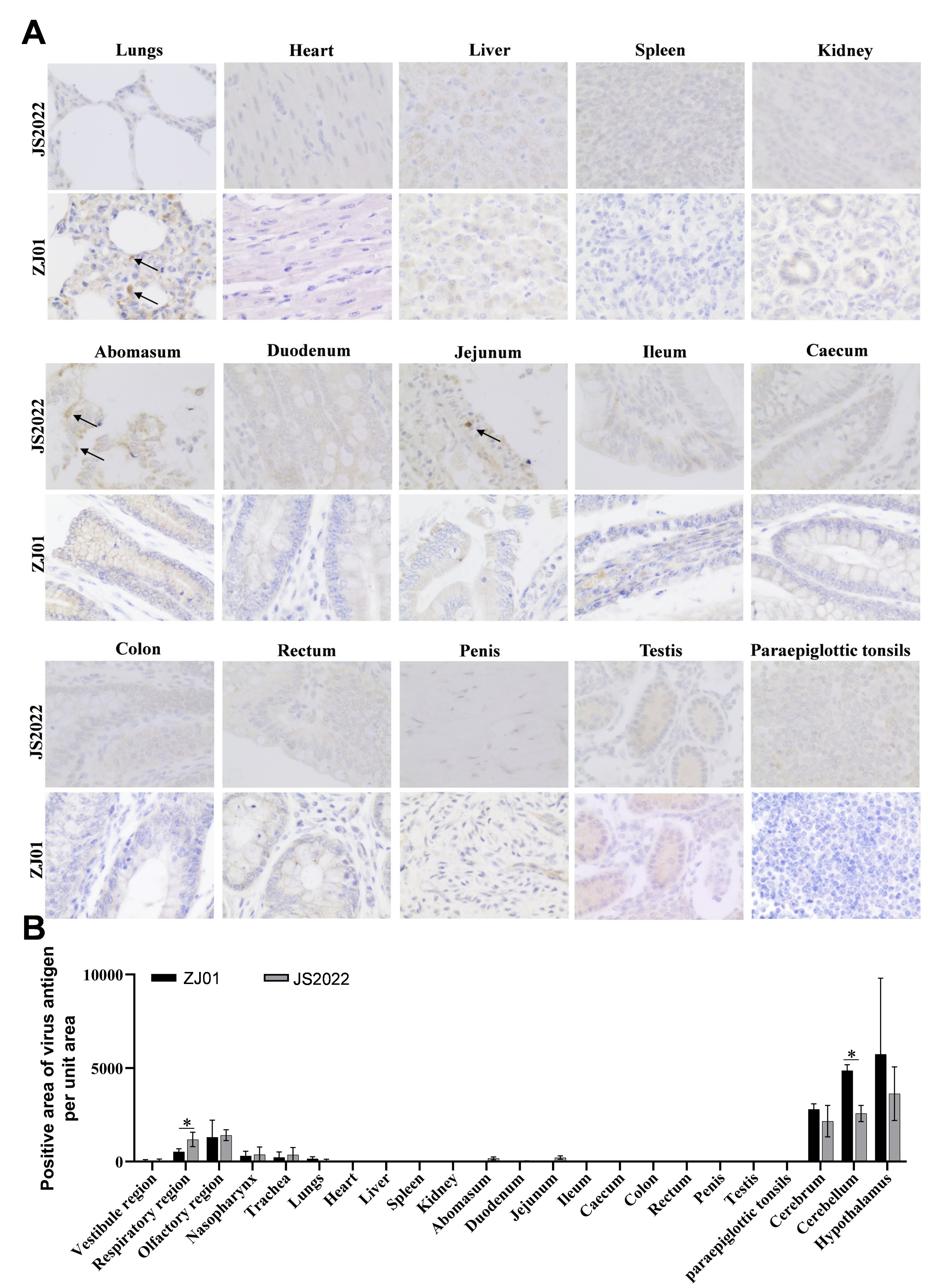


**Supplementary Figure 1. The localization of viral antigens in different organs of calves**

(A) Immunohistochemistry (IHC) analysis of Lungs, Heart, Liver, Spleen, Kidney, Abomasum, Duodenum, Jejunum, Ileum, Caecum, Colon, Rectum, Penis, Testis and Paraepiglottic tonsils with the mouse anti-PRV gB monoclonal antibody, brown positive particles (black arrows), original magnification ×400. (B) The positive area of virus antigen was counted from sections in a unit area (40×).


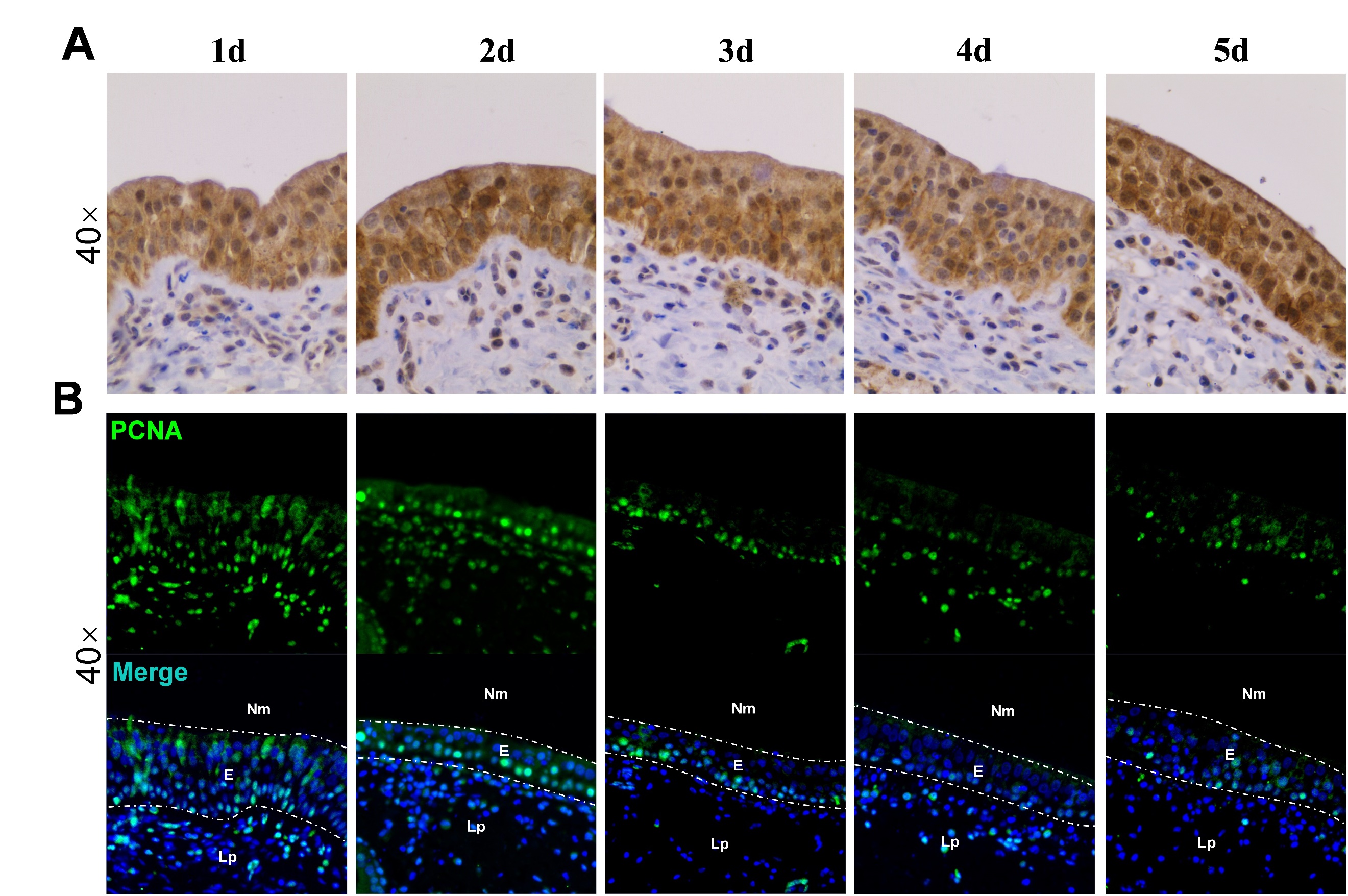


**Supplementary Figure 2. The establishment of bovine nasal mucosal explant culture models**

(A) The Claudin1 was used to evaluate the integrity of the epithelial barrier by immunohistochemistry. (B) The expression characteristics of PCNA were observed by immunofluorescence. E: Epithelium, Lp: Lamina propria, Nm: Nasal meatus.
